# Case of Encysted Stone

**Published:** 1844-02-24

**Authors:** Robert Storrs

**Affiliations:** Doncaster


### CASE OF ENCYSTED STONE.

By Robert Storrs, Esq., Doncaster.

Dec. 11, 1842. John Pearl, aged twenty-three, a pale, feeble, and emaciated young man, was admitted into the infirmary of the Doncaster union, about two months ago. He had been suffering from symptoms resembling those of stone from the time of his being eight years of age. He had, however, been apprenticed to a shoemaker, was married eighteen months since, and has now a child nearly six months old. About half a year ago he was a patient of the York County Hospital, when he was supposed to be laboring under disease of the bladder; from thence he was removed to this town, and after remaining in lodgings for some time was admitted into the union house with the following symptoms:—

Extreme pain in the region of the bladder, general uneasiness in the pelvis and hips, and an aching or itching pain at the glans penis. These pains are mitigated rather than increased by motion. The urine does not stop suddenly; it comes away in a tolerable stream, except when the pain is more than usually severe, and is then forced away involuntarily. The urine is copious but fœtid, and contains on standing a large portion of purulent matter. Has been sounded several times, but a stone cannot be detected, nor is there anything to be discovered unusual from an examination per anum. Both of these examinations, however, give him great pain. A great variety of treatment had been employed in the York Hospital, and he left there with his symptoms somewhat abated, but since he came under my care he has derived no benefit except from hot fomentations and strong opiates.

A few days after these symptoms were noted, a large discharge of blood took place with the urine, after which he gradually sunk and died.

Dec. 2. The day following I made a post-mortem examination of the body, and the following appearances were observed:—The body was more attenuated than I think I ever observed one to be, even in consumption, and the omentum quite void of fat. The bladder was divided into two portions by a septum; the upper, which contained the fundus, and the larger part of the right half of the bladder, formed a sac, in which a stone of the triple phosphate species was contained, about an ounce in weight, and of a cylindrical shape, but which did not nearly fill the whole of the containing sac. The entire of the mucous membrane of this portion, as well as of the lower division, which alone contained the urine was very much thickened; the whole bladder, indeed, was very much thicker than usual. The ureters were each as thick as a little finger, blueish in aspect, and containing, as well as the kidneys, which were also considerably enlarged, a large quantity of purulent matter. All the other organs, both of the chest and abdomen, were in a healthy state, except the descending colon, which was very much contracted for the space of three or four inches.—*Prov. Med. Jour.* Oct. 7, 1843.

